# Editorial: The liver as an endocrine organ: Hepatokines and ketone bodies, novel hormones to be acknowledged

**DOI:** 10.3389/fendo.2022.1117773

**Published:** 2023-01-04

**Authors:** Renata Risi, Catherine Postic, Mikiko Watanabe

**Affiliations:** ^1^ Department of Experimental Medicine, Section of Medical Pathophysiology, Food Science and Endocrinology, Sapienza University of Rome, Rome, Italy; ^2^ Université de Paris, Institut Cochin, CNRS, INSERM, Paris, France

**Keywords:** hepatokines, liver, obesity, NAFLD, ketones

The liver is the central regulator of metabolism homeostasis and disruption, capable of secreting hundreds of molecules with autocrine, paracrine, and endocrine effects. However, the complex interplay of these molecules in the regulation of metabolic diseases, as well as their possible application in clinical routine, are not well defined yet. This Research Topic gathered different contributions highlighting the role of novel or very well-known hepatokines in the pathogenesis of metabolic syndrome and inflammation, as well as obesity-associated comorbidities.

New and old hepatokines are largely implicated in the regulation of liver health. With regards to NAFLD pathogenesis, Iacob and Iacob reviewed the complex and coordinated role of different hepatokines/adipokines interplaying in this process. Moreover, they focused on HIV, HBV, and antiretroviral therapy-associated NAFLD, also pointing out what is known about the breakdown of the aforementioned hepatokines/adipokines axis in these infections. With regards to liver health, Szczepańska et al. focused, for the first time, on the physiological regulation of the hepatoprotective hepatokine FGF21 by thyroid hormones. They observed a transient FGF21 rise during rapid-onset hypothyroidism, shedding light on how in physiological conditions of increased hepatic lipogenesis, such as in cases of transient hypothyroidism, FGF21 rise may act as a compensatory mechanism to protect the liver against excessive triglyceride accumulation, further confirming other studies in the field (1, 2). Indeed, it was previously reported that FGF21 is increased in NAFLD and decreases with liver health improvement upon weight loss (3).

If understanding the mechanisms underlying liver disease is important, the challenge of our century is the identification of feasible and cheap biomarkers to stratify the population at higher risk of fibrosis and cancer. In this regard, the second original article included in our Research Topic (Zhu et al.) found that higher serum hsCRP levels in an obese Chinese population were associated with increased risk of MAFLD, and they correlated positively with the severity of liver steatosis and fibrosis, diagnosed with the non-invasive method FibroScan. This finding suggested that hsCRP can be used as a potential biomarker to monitor and predict MAFLD severity among subjects with obesity, and it may be of help to address patients to liver biopsies in clinical routines. Several biomarkers have been proposed recently and many are promising, although only a few were validated, and more research is warranted in this regard (4).

However, the complex spectrum of molecules secreted by the liver in health and disease can also act on other organs to contribute to obesity-associated systemic metabolic disruption and comorbidities. In this regard, Ren et al. proposed a more adipocentric view of the hepatokines/adpokines/miokines axis, having reviewed the anti-inflammatory and pro-inflammatory properties of 30 organokines in the pathogenesis of adipose tissue inflammation, a hallmark of metabolic impairment in obese patients. They summarized in a clear and interesting figure how these molecules regulate AT inflammation acting on adipocytes and adipose tissue macrophages as target cells. Finally, He et al. performed a metanalysis and meta-regression on 34 studies evaluating the correlation of serum/plasma concentrations of the well-known hepatokine IGF-1 with Obstructive Sleep ApneaHypopnea Syndrome (OSAHS) diagnosis and severity. They found considerably lower plasma and serum IGF-1 concentrations in patients with OSAHS compared with control, and these were negatively correlated with the severity of the disease. This, on the one hand, suggests a pathophysiological implication of the GH-IGF1 axis in the OSAHS pathogenesis, but on the other hand, it suggests the possible use of serum IGF1 levels as a biomarker for OSAHS severity.

Ketone bodies are molecules produced by the liver as a result of decreased dietary carbohydrate intake. Beyond being the main fuel in these conditions, they exert endocrine actions that allow them to be identified as proper hormones. Indeed, very low carbohydrate diets, inducing nutritional ketosis, have shown, on the one hand, to effectively improve NAFLD (5), and, on the other hand, to modulate the immune system (6, 7), among many other pleiotropic effects (8).

In summary, the liver is an endocrine organ producing several molecules with pleiotropic actions, and whose actions are only partially known. In this Research Topic we showed that there are several emerging hepatokines involved in the regulation of liver (Iacob and Iacob, Szczepańska et al.) and adipose tissue (Ren et al.) homeostasis in obesity. Moreover, it shed light on the possible application of well-known biomarkers (He et al.; Zhu et al.) in clinical routine for a better risk stratification of patients with obesity. We believe that the results derived from the works collected in this Research Topic represent further proof of how the identification of new and old metabolically relevant liver-derived molecules is essential to deeply understand the protective role of the liver in maintaining metabolic homeostasis and its contribution to systemic disruption.

## Author contributions

All authors listed have made a substantial, direct, and intellectual contribution to the work and approved it for publication.
